# Zooplankton abundance and environmental variables at weathership station India, 59 ^o^ N 19 ^o^ W, from 1974 to 1975

**DOI:** 10.12688/openreseurope.21298.1

**Published:** 2025-09-10

**Authors:** Xabier Irigoien

**Affiliations:** 1 AZTI - BRTA, Pasaia, Euskadi, Spain; 2 Ikerbasque, Bilbao, Basque Country, Spain

**Keywords:** Zooplankton, vertical distribution, North Atlantic, ocean time series

## Abstract

This dataset was collected between 1971 and 1975 at Weathership Station India (59°N, 19°W) in the North Atlantic Ocean. It comprises quantitative records of several zooplankton taxa— *Calanus finmarchicus*, *Metridia lucens*, *Pleuromamma robusta*, *Oithona* spp., *Oncaea* spp., and *Acartia* spp.—alongside measurements of phytoplankton abundance, temperature, salinity, chlorophyll *a* concentration, Secchi depth, and primary production. Sampling frequency and depth resolution varied by parameter. The dataset is organized into separate Excel files for each variable.

## Introduction

The vertical structure and transport processes in open-ocean ecosystems represent a significant knowledge gap in our understanding of global biogeochemical cycles ( Martin *et al*., 2020). Despite their importance, vertically resolved biological datasets from the open ocean remain scarce, primarily due to the logistical and financial constraints associated with long-term ship-based observations. Consequently, seasonal dynamics of vertical ecosystem structure are poorly characterized. While autonomous platforms are expected to address this limitation in the future, current biological data from such systems remain limited in scope and resolution.

Between the 1970s and 1990s, weather ships stationed in the open ocean provided continuous meteorological data for forecasting purposes. These vessels also offered unique opportunities for oceanographic sampling (e.g., Batchelder, 1985; Irigoien *et al*., 1998; Irigoien, 1999; Irigoien & Harris, 2006; Mackas *et al*., 1998). The data collected during these campaigns offer rare insights into seasonal variability in oceanic ecosystems as well as serving as historical baselines for climate change studies. However, much of this information remains difficult to access.

One such dataset was acquired at Weathership Station India (59°N, 19°W) between 1971 and 1975 ( Williams & Hopkins, 1971; Williams & Hopkins, 1975a). Portions of this dataset were later recovered under the Plankton Reactivity in the Marine Environment (PRIME) project and archived in the British Oceanographic Data Centre (BODC). However, the fragmented format—one file per species and sampling date—has hindered its usability. As part of the European Union Horizon 2020 project *Sustainable Management of Mesopelagic Resources* (SUMMER, Grant No. 817806), these data have now been consolidated into accessible formats. This paper presents the dataset and aims to promote its use in ecological related research.

## Materials and methods

Sampling was conducted from 1971 to 1974 at Weathership Station India, located approximately 260 nautical miles south of Iceland on the western slope between the Hatton Bank and the Iceland Basin, at a depth of ~2000 m. The region is characterized by a deep mixed layer during winter (September–March) due to strong wind forcing, followed by stratification during calmer periods. The sampling period was characterized by the passage of the great salinity anomaly (1968–1982, Dickson *et al*., 1988).

Sampling methodologies are detailed in Williams (1988) and Irigoien (2000). In brief, vertical distributions of copepods were assessed weekly from March to October using oblique tows with a Longhurst-Hardy Plankton Recorder (280 µm mesh). Tows were designed to sample every 10 m from 500 m to the surface. Hydrographic profiles (temperature and salinity) were obtained approximately every five days at 19 discrete depths from the surface to 2000 m. Chlorophyll *a* concentration was measured from water samples collected at 10 depths (0–200 m) approximately every two days. According to the records the method used to measure Chl *a* was described by Adams and Baird in *Annls. biol., Copenh.,* 26: 113–114. We could not find that complete description, but after further research Chl *a* was most likely measured using the Yentsch and Menzel (1963) method. Basically, Samples for analysis of photosynthetic pigments were collected by filtration through Whatman GF/C glass fiber filters, frozen until assayed, extracted with 90% v/v acetone and measured by fluorometry using and Turner fluorometer). Primary production was measured through ^14^C incubations following Strickland and Parsons (1968) where a known amount of radioactive carbonate ^14^CO _3_ is added to the sea water sample and incubated for a determined period. After incubation samples are filtered, washed and dried, and the radioactivity from the ^14^CO _3_ assimilated by phytoplankton measured using a scintillation counter. We could not find the detailed methodology for the phytoplankton taxonomic identification. However, it can be assumed that samples were fixed in 1% lugol and counted under an inverted microscope after settling Utermohl technique, following an approach like Holligan and Harbour (1977). Secchi ( Secchi, 1864) depth is a measure of water clarity, determined by the depth at which a black and white disk (Secchi disk) disappears when lowered into the water. The Secchi disk, typically a 30 cm white disk with alternating black and white quadrants, is attached to a rope. The observer records the depth at which the disk can no longer be seen is recorded as the Secchi depth. It is a widely used technique, but attention should be paid in the file to the notes about cable angles and state of the sea that bias the measurements.

## Data description

The dataset is publicly available via Zenodo ( https://doi.org/10.5281/zenodo.15862644), ( Irigoien, 2025) under a Creative Commons Attribution 4.0 International license. It includes six Excel files corresponding to the following parameters: chlorophyll *a* (Chlorophyll a.xlsx), zooplankton taxonomic abundance (India all copepods m3.xlsx), phytoplankton taxonomic abundance (Phytoplankton.xlsx), primary production (Primary production.xlsx), Secchi depth (SECCHI depth.xlsx), and temperature and salinity (Temperature and Salinity.xlsx). Although historical records indicate that additional data (e.g., nutrients, euphausiids, amphipods, jellyfish) were collected ( Williams & Conway, 1981; Williams & Hopkins, 1975b; Williams & Lindley, 1982; Williams & Robins, 1981), these data was not recovered by the PRIME project.

The zooplankton dataset includes abundance data for *Calanus finmarchicus*, *Metridia lucens*, *Pleuromamma robusta*, *Oithona* spp., *Oncaea* spp., and *Acartia* spp. Users should exercise caution when interpreting *Acartia* data, as inconsistencies exist between total and stage-specific counts due to changes in stage classification across original files. Similarly, phytoplankton data should be interpreted with care. Although the methodology is undocumented, it is presumed that samples were preserved with Lugol’s solution and analyzed microscopically following sedimentation, as described in Irigoien *et al*. (2000). The accuracy of species-level identification may vary depending on the analyst, and it is unclear whether all samples were processed by the same individual. Therefore, while data for major groups (e.g., diatoms, dinoflagellates, coccolithophores) are likely reliable, species-level presence/absence data should be treated with caution.

## Ethics and consent

Ethical approval and consent were not required.

## Data Availability

The dataset is publicly available via Zenodo Zenodo: Weathership India 1971–1975 mesozooplankton and additional environmental data ( https://doi.org/10.5281/zenodo.15862644), ( Irigoien, 2025). It includes six Excel files corresponding to the following parameters: Chlorophyll a.xlsx: Chlorophyll *a* measurement in mg Chl *a* m ^-3^, India all copepods m3.xlsx: zooplankton taxonomic abundance ( *Calanus finmarchicus*, *Metridia lucens*, *Pleuromamma robusta*, *Oithona* spp., *Oncaea* spp., and *Acartia* spp.) in ind m ^-3^ Phytoplankton.xlsx: Phytoplankton taxonomic abundance in cell l ^-1^ Primary production.xlsx: primary production in C14 uptake mg C.m ^-3^.day ^-1^ SECCHI depth.xlsx: Secchi depth in m Temperature and Salinity.xlsx: temperature and salinity in °C and ppt. Data is available under the terms of the Creative Commons Attribution 4.0 International license (CC-BY 4.0).
