# Supplementary material for: Zooplankton abundance and environmental variables at weathership station India, 59 o N 19 o W, from 1971 to 1975
Source: Open Res Eur. 2025 Oct 7;5:277. Originally published 2025 Sep 10. [Version 2] doi: 10.12688/openreseurope.21298.2 (PMC12605589; doi:10.12688/openreseurope.21298.2)
Supplement: Peer review history

# Reviewer report: Reviewer response for version 2

**DOI:** 10.21956/openreseurope.23311.r61967

**Published:** 2025-11-22

**Authors:** Inma Herrera

**Affiliations:** 1 Universidad de Las Palmas de Gran Canaria, Telde, Spain

The article is a Data Note describing a historical dataset of zooplankton abundance and environmental variables collected at *Weathership Station India (59°N, 19°W)* in the North Atlantic between 1971 and 1975. The dataset includes chlorophyll *a*, primary production, temperature, salinity, Secchi depth, phytoplankton abundance and abundance of six copepod taxa ( *Calanus finmarchicus, Metridia lucens, Pleuromamma robusta, Oithona spp., Oncaea spp., Acartia spp.*). As stated in the article:

 “This dataset was collected between 1971 and 1975 at Weathership Station India…” The data have been digitized and organized into seven Excel files, and made publicly available on Zenodo under a CC-BY 4.0 license:

 “The dataset is publicly available via Zenodo… It includes seven Excel files corresponding to the following parameters…”

 The article explains the sampling methodologies, the structure of the dataset, and known limitations (e.g., incomplete phytoplankton taxonomy, gaps in zooplankton counts). An amendment section details corrections made after reviewer feedback. 

  Is the rationale clearly described? — **Yes**

 The need for this dataset is well justified. The paper emphasizes the rarity of long-term, vertically resolved biological datasets in open ocean systems and contextualizes the sampling period with the Great Salinity Anomaly:

 “Historical datasets including seasonality covering several years are rare and hence valuable.”

 The article clearly motivates why recovering and organizing these data has scientific value.

 Are the protocols appropriate and technically sound? — **Yes**

 The sampling instruments and techniques are appropriate and well referenced. Zooplankton sampling was carried out with Longhurst-Hardy Plankton Recorder tows and hydrographic profiles were obtained to 2000 m depth. Chlorophyll *a* and primary production methods are supported by literature.

 The author is transparent regarding uncertainties in sample processing (e.g., phytoplankton taxonomy, copepod stage inconsistencies), which strengthens the credibility of the dataset.

 Are sufficient methods provided to allow replication? — **Partly**

 While most methods are described, **some methodological details are missing or incomplete**, particularly for:

1. **Zooplankton preservation and taxonomic counting protocol**
  The paper does not describe how samples were preserved and analyzed. One reviewer also requested details about sample treatment.
2. **Depth of zooplankton sampling**
  The article associates *mid-depth* with the Longhurst-Hardy sampling but does not define explicit min–max depth per tow in the dataset files. The paper states:
  “Sampling ranges were not available in the original files, only the mid-depth…”
3. **Phytoplankton identification**

The methodology is speculative (“it can be assumed…”), not fully documented.

1. **Interpretation of empty cells vs. zero values**
  Empty cells in phytoplankton and zooplankton spreadsheets need clarification (0 abundance vs. not measured)

*Recommendation (improvement but not blocking scientific validity):*

- Add explicit statements in Methods and/or metadata indicating:
  - preservation method and counting approach for net samples
  - definition of *mid-depth* and assumed depth range sampled
  - specify whether blank cells mean “0 detected” or “no data”

Are datasets clearly presented and accessible? — **Partly**

 The dataset is accessible (Zenodo; Excel format), and the author already uploaded a corrected file including day, month, year, and hour.

 However, two issues reduce usability:

1. **Abbreviated taxonomic names in phytoplankton file**
  The article acknowledges that abbreviations cannot be validated and warns users:
  “Abbreviations have been left as in the original files and again caution is requested…”
2. **Column headings and language inconsistencies in some Excel files**
3. **Missing metadata for depth intervals in zooplankton tows**

*Recommendation:*

 Add a metadata sheet in each Excel file indicating:

-
  - explanation of column names (English)
  - taxonomic abbreviation key (even if incomplete)
  - meaning of empty cells vs. zeros

These improvements increase data reusability for future users.

 This is a scientifically valuable data note. The work is technically sound, the rationale is clear, and the dataset fills an important gap in long-term open-ocean ecological records.

 Only minor clarifications are needed to maximize dataset usability. None affect the scientific integrity of the dataset.

Are sufficient details of methods and materials provided to allow replication by others?

Partly

Is the rationale for creating the dataset(s) clearly described?

Yes

Are the datasets clearly presented in a useable and accessible format?

Partly

Are the protocols appropriate and is the work technically sound?

Yes

Reviewer Expertise:

Zooplankton ecology / Oceanography / Marine environmental data analysisThe dataset is valuable, the rationale is clearly described, and the work is technically sound. However, some methodological details (e.g., sample preservation and taxonomic counting procedures) are only partially reported, and certain data files require additional clarification (empty cells vs. zeros, depth intervals, phytoplankton abbreviations). These points do not compromise the scientific validity of the dataset but should be addressed to maximize reusability.

I confirm that I have read this submission and believe that I have an appropriate level of expertise to confirm that it is of an acceptable scientific standard, however I have significant reservations, as outlined above.

# Reviewer report: Reviewer response for version 2

**DOI:** 10.21956/openreseurope.23311.r61970

**Published:** 2025-11-11

**Authors:** Camilla Svensen

**Affiliations:** 1 UiT The Arctic University of Norway, Tromsø, Norway

This data note entitled «Zooplankton abundance and environmental variables at weathership station India, 59 N 19 W, from 1971 to 1975 (version 2; peer review: 1 approved) submitted by X. Irigoien, provide data on Chlorophyll a, primary production, temperature and salinity, secchi depth and seven copepod taxa collected between 1971 and 1975 at weathership Station India. The dataset is organized in six Excel files - one for each variable, including one updated excel file for zooplankton abundances.

 Historical datasets including seasonality covering several years are rare and hence valuable. This dataset is especially interesting because it covers a period characterized by the great salinity anomaly.

 I noted some inconsistencies in some of the excel files, and some needs for clarification both in the data description and excel files. My specific comments are provided below.

Materials and methods

- Add a sentence on sample treatment for the net tows, e.g. preservation method and counting procedure if this information is available.
- Abundances of seven copepod taxa are included in the dataset, but likely there were more species present in the original samples. If possible, a short note on the selection of taxa would be clarifying. For instance, were these the most abundant taxa, or were they selected for monitoring due to other reasons?
- It could be mentioned that the mesh-size of 280 µm is rather large and is likely to under-sample small taxa and young developmental stages of copepods.
- Please add information about the sampling depths of zooplankton, and explain what is meant by mid-depth.
- Add abbreviation after the first mentioning of Chlorophyll a (Chl a)
- Last paragraph of methods section: remove parenthesis after «...Turner fluorometer).»

Datasets

 India_all_copepods_m3_corrected

- Adding depth-intervals (e.g. two more columns with minimum and maximum depths) for the zooplankton tows, in addition to mid-depths would be useful.
- Headings of columns should be in English language.
- For some of the sampling dates, the dataset indicates 0 ind m3 for the following copepod taxa: *Acartia* spp. for samplings after 31.03.1975, *Oithona* sp. for samplings on 30.07.1974, 31.08.1974, 30.09.1974, 31.03.1975, 23.04.1975, 26.05.1975, 06.06.1975, *Oncaea* sp. for samplings on 27.06.74, 30.07.1974, 31.08.1974, 30.09.1974,31.03.1975,23.04.1975,26.05.1975, 06.06.1975. Is there any explanation for the absence to be added in the methods section? Were these taxa not enumerated on these dates (hence NA) or were they not present in samples.
- There are two sampling points on 27.06.1974 (19:46 and 23:52) – is this correct?
- *Metridia lucens*: for sampling dates 26.07.1973 and 11.09.1974 no data are presented (empty cells). Should it be 0 ind m3 or NA?

Phytoplankton

-  There are some empty cells in the file. Should it be 0 or NA?

Primary production

- Spelling error in column D (uptake)

Secci depth

- Regarding the parameter “state of the sea” – is there an explanation for the scale that could be included in the methods section? At least it should be indicated if 1 means rough or calm conditions and if 10 is the maximum.

Are sufficient details of methods and materials provided to allow replication by others?

Partly

Is the rationale for creating the dataset(s) clearly described?

Yes

Are the datasets clearly presented in a useable and accessible format?

Partly

Are the protocols appropriate and is the work technically sound?

Yes

Reviewer Expertise:

Zooplankton ecology and functional biodiversity, secondary production and the role of zooplankton for the biological carbon pump.

I confirm that I have read this submission and believe that I have an appropriate level of expertise to confirm that it is of an acceptable scientific standard.

# Reviewer report: Reviewer response for version 2

**DOI:** 10.21956/openreseurope.23311.r61767

**Published:** 2025-10-16

**Authors:** Taketoshi Kodama

**Affiliations:** 1 The University of Tokyo, Bunkyo, Japan

I'm satisfied with the revision. On the database, I clearly understand the situation.

Are sufficient details of methods and materials provided to allow replication by others?

Yes

Is the rationale for creating the dataset(s) clearly described?

Yes

Are the datasets clearly presented in a useable and accessible format?

Partly

Are the protocols appropriate and is the work technically sound?

Yes

Reviewer Expertise:

Oceanography

I confirm that I have read this submission and believe that I have an appropriate level of expertise to confirm that it is of an acceptable scientific standard.

# Reviewer report: Reviewer response for version 1

**DOI:** 10.21956/openreseurope.23034.r60456

**Published:** 2025-09-26

**Authors:** Taketoshi Kodama

**Affiliations:** 1 The University of Tokyo, Bunkyo, Japan

The manuscript entitled “Zooplankton abundance and environmental variables at weather station India, 59°N 19°W, from 1974 to 1975” by Xabier Irigoien et al. was submitted as a data note. The dataset is highly valuable, and I therefore recommend indexing of this data note after revision. Since the dataset itself constitutes the results, and no formal results or discussion sections are expected, my comments are limited to minor revisions regarding consistency and formatting.

 1.    Title and text consistency

     •    Please revise the title. I believe the dataset covers the years 1971 to 1975, not 1974 to 1975.

     •    Similarly, the first sentence of the Materials and Methods should be corrected from “Sampling was conducted from 1971 to 1974” to “Sampling was conducted from 1971 to 1975.”

 2.    Zenodo files

     •    The files in Zenodo should be revised for consistency. In particular, columns for year, month, day, and time should be included in every file. For example, “9h” alone is insufficient information. Harmonized datasets with standardized column headers will greatly facilitate future meta-analyses.

     •    In the file India all copepods m3.xlsx, please clarify the meaning of “mid-depth.” The statement “Tows were designed to sample every 10 m from 500 m to the surface” does not provide sufficient information for interpretation. If sampling ranges were recorded, they should be explicitly noted in the Excel file.

     •    Please avoid using abbreviations for genus names. For example, “Cerat. Furca” may refer to Ceratium furca, but this is unclear. Similarly, “Boto” may indicate Boto spp. (given that Fragilaria spp. are mentioned). If abbreviations are intentional, please provide an explanation.

 In sum, the dataset represents a highly valuable long-term record, and with these minor corrections, it will provide a robust and reusable resource for the community.

Are sufficient details of methods and materials provided to allow replication by others?

Yes

Is the rationale for creating the dataset(s) clearly described?

Yes

Are the datasets clearly presented in a useable and accessible format?

Partly

Are the protocols appropriate and is the work technically sound?

Yes

Reviewer Expertise:

Oceanography

I confirm that I have read this submission and believe that I have an appropriate level of expertise to confirm that it is of an acceptable scientific standard, however I have significant reservations, as outlined above.

# Response

**Published:** 2025-10-01

**Authors:** Xabier Irigoien

**Affiliations:** AZTI Foundation, Pasaia, Euskadi, Spain

1.    Title and text consistency

     •    Please revise the title. I believe the dataset covers the years 1971 to 1975, not 1974 to 1975. *Corrected in the new version*

     •    Similarly, the first sentence of the Materials and Methods should be corrected from “Sampling was conducted from 1971 to 1974” to “Sampling was conducted from 1971 to 1975.” *Corrected in the new version.*

 2.    Zenodo files

     •    The files in Zenodo should be revised for consistency. In particular, columns for year, month, day, and time should be included in every file. For example, “9h” alone is insufficient information. Harmonized datasets with standardized column headers will greatly facilitate future meta-analyses. *A new file with additional 4 columns has been created and added to Zenodo. The new columns contain day, month, year and hour (when available). Some other files without information have been cleaned out.The previous file has been maintained in zenodo as it has been downloaded by different people and I believe is important they can find the original file. The changes are reported in the new version.*

     •    In the file India all copepods m3.xlsx, please clarify the meaning of “mid-depth.” The statement “Tows were designed to sample every 10 m from 500 m to the surface” does not provide sufficient information for interpretation. If sampling ranges were recorded, they should be explicitly noted in the Excel file. *Sampling ranges were not available in the original files, only the mid-depth of the range. Because of the sampling design it can be assumed that the range is more or less 5 m above and below the mid depth. But the detailed information was not available.*

     •    Please avoid using abbreviations for genus names. For example, “Cerat. Furca” may refer to Ceratium furca, but this is unclear. Similarly, “Boto” may indicate Boto spp. (given that Fragilaria spp. are mentioned). If abbreviations are intentional, please provide an explanation. *The abbreviations is the only information available in the original file. They could be interpreted but as it is not possible to validate with the originator for the dubious cases I think is better to keep the original information and reinforce the message about being cautious using the species level. The alternative would be to eliminate the phytoplankton file, but I believe that it contains valuable information although limited at the taxonomic level.*

 In sum, the dataset represents a highly valuable long-term record, and with these minor corrections, it will provide a robust and reusable resource for the community.
